# In vivo three-dimensional FD-OCT visualization of microvascular structures consistent with vasa vasorum in a conventionally harvested saphenous vein graft

**DOI:** 10.1186/s44215-026-00276-z

**Published:** 2026-09-15

**Authors:** Akira Sugaya, Mutsumi Mizoguchi, Masanori Nakamura, Koji Kawahito

**Affiliations:** 1 Division of Cardiovascular Surgery, Jichi Medical University School of Medicine, 3311-1 Yakushiji, Shimotsuke, Tochigi 3290498 Japan; 2 Department of Electrical and Mechanical Engineering, Nagoya Institute of Technology, Aichi, Japan

**Keywords:** Coronary artery bypass grafting, Saphenous vein graft, Vasa vasorum, Optical coherence tomography, No-touch technique

## Abstract

**Background:**

The no-touch (NT) saphenous vein graft (SVG) technique improves long-term graft patency. Proposed mechanisms include maintenance of adventitial and perivascular structures and avoidance of high-pressure distension. Whether adventitial structures consistent with VV remain detectable in carefully harvested conventional SVGs without a thick tissue pedicle remains uncertain.

**Case presentation:**

A 75-year-old woman underwent aortic valve replacement and coronary artery bypass grafting. The SVG was harvested using a carefully performed conventional technique that retained an approximately 1–2 mm rim of surrounding adipose tissue and protected the adventitia. Graft dilation was performed using cardiopulmonary bypass arterial-line pressure (approximately 100 mmHg), avoiding manual high-pressure distension. Two weeks postoperatively, frequency-domain optical coherence tomography (FD-OCT) demonstrated low-signal tubular structures within the adventitial layer. Three-dimensional reconstruction showed longitudinal continuity of structures morphologically consistent with vasa vasorum externae.

**Conclusions:**

This case demonstrates localized adventitial structures consistent with VV in a carefully harvested conventional SVG. Although functional patency could not be confirmed, the findings are hypothesis-generating and warrant further comparative investigation.

**Supplementary Information:**

The online version contains supplementary material available at https://doi.org/10.1186/s44215-026-00276-z.

## Background

The no-touch (NT) saphenous vein graft (SVG) technique has demonstrated superior long-term patency compared with conventional harvesting [1, 2]. Proposed mechanisms include preservation of perivascular adipose tissue and adventitial integrity, which may help maintain the vasa vasorum (VV), as well as avoidance of high-pressure distension, which may reduce endothelial and intimal injury [3–5]. Despite these advantages, harvesting the vein with a thick pedicle of surrounding tissue may increase leg wound complications and is not universally adopted [6, 7]. Because the VV network is primarily located within the adventitia and adjacent perivascular tissue [8], careful protection of the adventitia may help retain elements of this microvascular architecture. We report in vivo FD-OCT findings demonstrating adventitial microvascular structures consistent with VV in an SVG harvested using a carefully performed conventional technique and physiologic-pressure dilation.

## Case presentation

A 75-year-old woman with severe aortic stenosis and multivessel coronary artery disease underwent aortic valve replacement and coronary artery bypass grafting. The SVG was harvested from the right lower leg using Metzenbaum scissors and an electrosurgical unit (VIO 200 D; Erbe Elektromedizin GmbH, Tübingen, Germany) in the DRY CUT and FORCED COAG modes, both set at 30 W. Dissection was performed under direct visualization of the external surface of the vein wall, with an approximately 1–2 mm rim of surrounding adipose tissue retained to avoid injury to the adventitial layer. Side branches were ligated and divided approximately 1–2 mm from the SVG trunk to minimize injury to the adventitia and its associated microvascular structures. The harvesting procedure is shown in Additional File 1. Following harvest, the graft was gently dilated using arterial-line pressure from the cardiopulmonary bypass circuit (approximately 100 mmHg), avoiding manual syringe distension. The SVG was subsequently used for coronary bypass grafting.

**Fig. 1:**
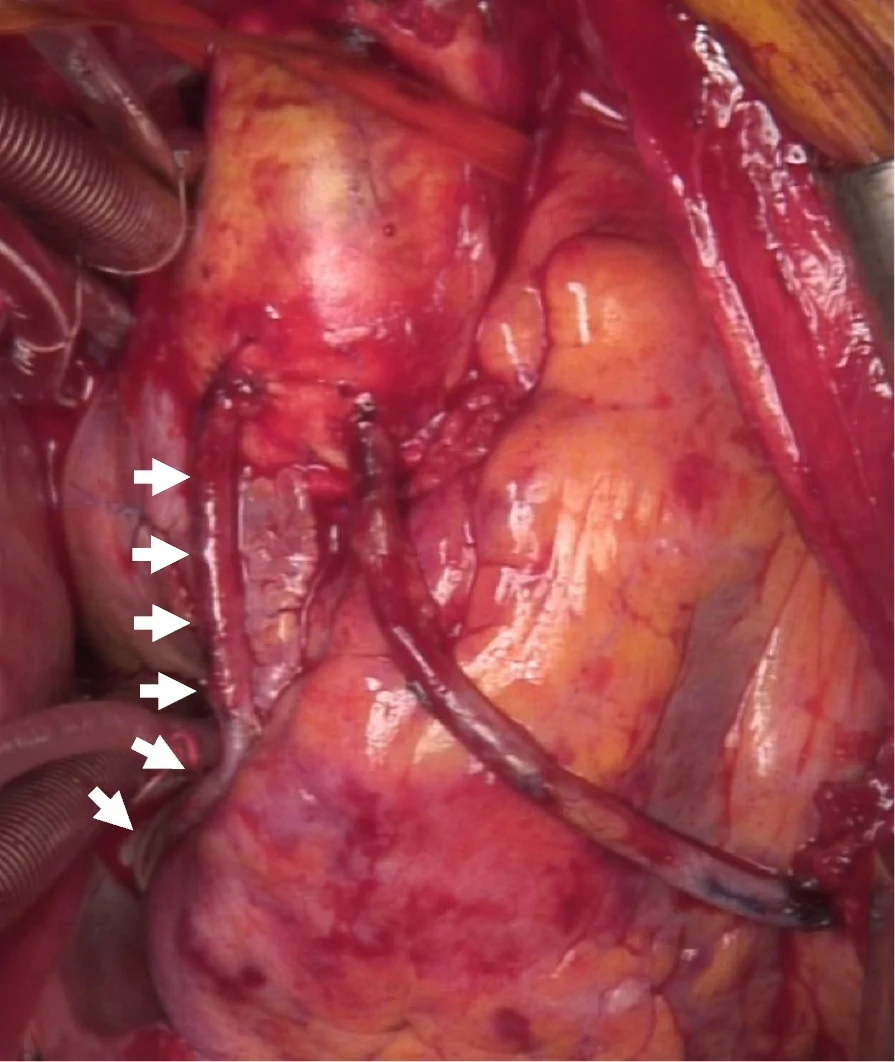
Intraoperative appearance of the saphenous vein graft prepared with refined conventional harvesting. The graft is harvested with preservation of the adventitial layer and minimal surrounding adipose tissue, avoiding a thick pedicle. The arrow indicates the segment analyzed by FD-OCT

Routine postoperative coronary angiography was performed 2 weeks after surgery. FD-OCT imaging was obtained using a Dragonfly OpStar imaging catheter and SJM FD-OCT Imaging System (Abbott Cardiovascular, Chicago, IL, USA). FD-OCT imaging was performed over an approximately 60-mm proximal segment of the graft. Of this segment, an approximately 10-mm region containing structures consistent with VV was selected for three-dimensional reconstruction (Fig. 1). Structures consistent with VV were defined as signal-poor tubuloluminal structures identified on serial cross-sectional and longitudinal FD-OCT images and located within the adventitial or immediately perivascular layer, according to previously reported and histologically validated OCT criteria [9].

For three-dimensional analysis, FD-OCT images were processed using the open-source software platform 3D Slicer. Signal-poor tubuloluminal structures located within the adventitia or immediately perivascular layer and traceable across consecutive FD-OCT frames were selected for segmentation. Their luminal regions were manually delineated frame by frame, without automated or semi-automated segmentation methods such as intensity thresholding or region growing. The resulting segmentations were independently reviewed by two cardiovascular surgeons. Finally, the segmented structures were reconstructed into a three-dimensional model and exported in stereolithography format.

**Fig. 2:**
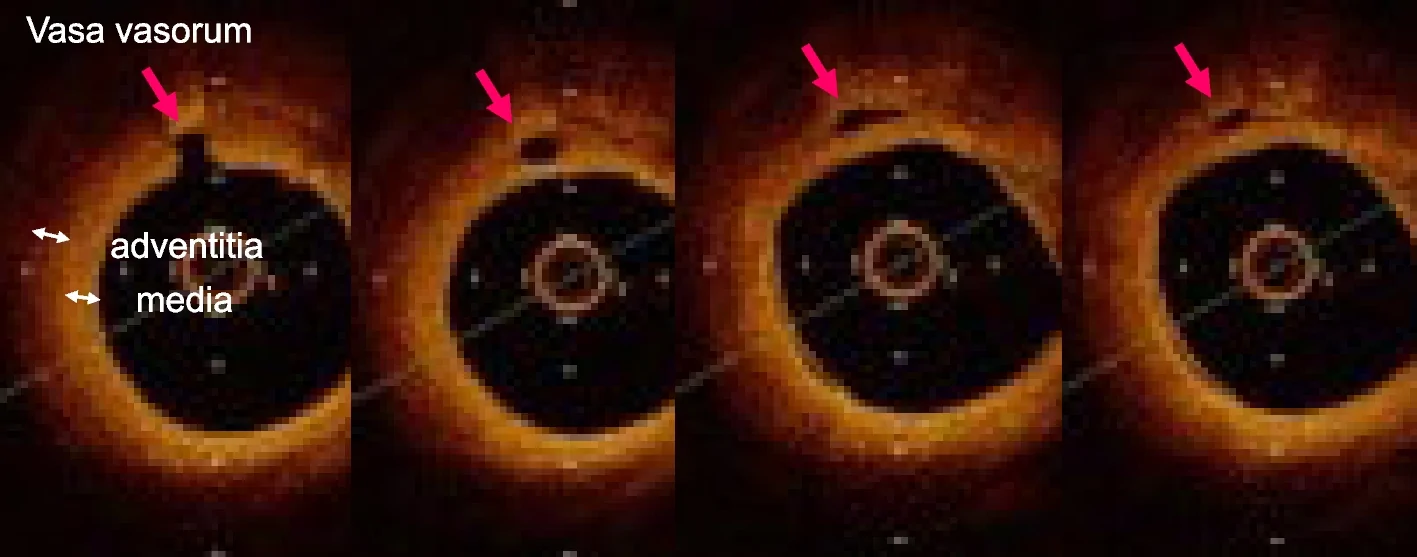
Cross-sectional FD-OCT images demonstrating adventitial microvascular structures consistent with vasa vasorum. Signal-poor tubuloluminal structures are observed within the adventitial layer in close association with a graft side branch. Their location and morphology were considered most compatible with relatively proximal vasa vasorum externae or their feeding vessels. The structure shown in Fig. 2 is visualized in Additional File 2 between 1 min 02 s and 1 min 05 s at approximately the 11 to 12 o’clock position. A separate structure consistent with vasa vasorum externae is also visualized between 57 s and 1 min 00 s, extending from approximately the 8 to 6 o’clock position

**Fig. 3:**
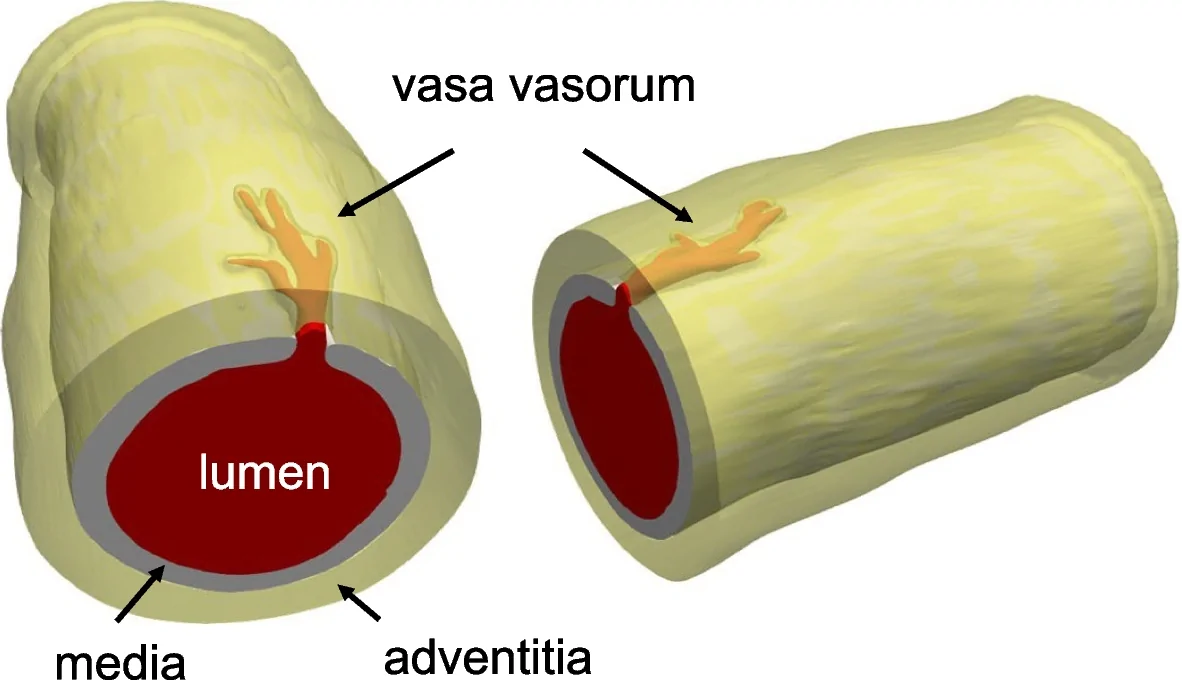
Three-dimensional FD-OCT reconstruction of the saphenous vein graft. Three-dimensional reconstruction demonstrates longitudinal continuity of the adventitial tubular structures along the graft. Their spatial distribution is consistent with side-branch-associated adventitial tubular structures compatible with VV-related structures

FD-OCT demonstrated several signal-poor tubular structures within the adventitial layer in close association with graft side branches. These structures were visualized on serial cross-sectional and longitudinal images and extended longitudinally along the graft (Fig. 2, Additional File 2). Three-dimensional reconstruction demonstrated longitudinal continuity within the adventitial region (Fig. 3). Their morphology and distribution were considered consistent with VV. Representative FD‑OCT findings of conventional and no‑touch SVGs are shown in Additional File 3. These observations are supported by our previous detailed investigation of FD‑OCT characteristics in both graft types [10].

## Discussion

This report provides in vivo FD-OCT visualization of localized adventitial microvascular structures consistent with VV in a carefully harvested conventional SVG. In this case, only an approximately 1–2-mm rim of surrounding adipose tissue was retained, yet longitudinally continuous tubular structures were detected within the adventitial layer. These findings suggest that meticulous preservation of the adventitia may help retain adventitial structures consistent with VV.

Vasa vasorum have been anatomically classified as vasa vasorum interna, vasa vasorum externa, and venous vasa vasorum [11]. The prominent tubular structure shown in Fig. 2 was larger than the fine microvascular structures previously observed in no-touch SVGs [10]. Its close association with a graft side branch and longitudinal course within the adventitial layer make it morphologically most compatible with a relatively proximal component of the vasa vasorum externa or a feeding vessel of this network. Additional serial FD-OCT images also showed other adventitial tubuloluminal structures, supporting the presence of VV-related structures beyond the representative finding shown in Fig. 2.

Several limitations should be acknowledged. FD-OCT provides morphological information only and cannot directly confirm blood flow, functional patency, or histological identity. Because pre-harvest or baseline imaging was not available, true preservation of pre-existing VV could not be demonstrated. A residual side-branch remnant, another side-branch-related vascular structure, or a disconnected anatomical remnant could not be completely excluded. Although the persistence of these tubuloluminal structures 2 weeks after surgery may suggest that they were not immediately obliterated, this observation does not establish functional perfusion or biological activity. It therefore remains uncertain whether these structures contributed to oxygen and nutrient supply of the graft wall.

In conclusion, localized adventitial structures consistent with VV were demonstrated in a conventional SVG harvested with meticulous adventitial preservation. These findings are hypothesis-generating and require confirmation in comparative studies.

## Supplementary Information

Additional file 1.

Additional file 2.

Additional file 3.

## Data Availability

All relevant data are included in this article.

## Abbreviations

CABG: Coronary artery bypass grafting
FD-OCT: Frequency-domain optical coherence tomography
NT: No-touch
PAT: Perivascular adipose tissue
SVG: Saphenous vein graft
VV: Vasa vasorum

## References

[1] Souza DS, Johansson B, Bojö L, Karlsson R, Geijer H, Filbey D, et al. Harvesting the saphenous vein with surrounding tissue for coronary artery bypass grafting provides long-term graft patency comparable to the left internal thoracic artery: results of a randomized longitudinal trial. J Thorac Cardiovasc Surg. 2006;132:373–8. 16872965 10.1016/j.jtcvs.2006.04.002

[2] Samano N, Geijer H, Liden M, Fremes S, Bodin L, Souza D. The no-touch saphenous vein for coronary artery bypass grafting maintains patency, after 16 years, comparable to the left internal thoracic artery: a randomized trial. J Thorac Cardiovasc Surg. 2015;150:880–8. 10.1016/j.jtcvs.2015.07.027. 26282605

[3] Deng MX, Lia H, Lee G, Rahouma M, Di Franco A, Demetres M, et al. Angiographic patency of coronary artery bypass conduits: an updated network meta-analysis of randomized trials. Braz J Cardiovasc Surg. 2022;37:7–31. 36053998 10.21470/1678-9741-2022-0142 PMC9454285

[4] Dashwood MR, Anand R, Loesch A, Souza DS. Hypothesis: a potential role for the vasa vasorum in the maintenance of vein graft patency. Angiology. 2004;55:385–95. 15258684 10.1177/000331970405500405

[5] Loesch A, Dashwood MR. Vasa vasorum inside-out/outside-in communication: a potential role in the patency of saphenous vein coronary artery bypass grafts. J Cell Commun Signal. 2018;12:631–43. 30078142 10.1007/s12079-018-0483-1 PMC6235771

[6] Aldea GS, Bakaeen FG, Pal J, Fremes S, Head SJ, Sabik J, et al. The Society of Thoracic Surgeons clinical practice guidelines on arterial conduits for coronary artery bypass grafting. Ann Thorac Surg. 2016;101:801–9. 10.1016/j.athoracsur.2015.09.100. 26680310

[7] Deb S, Singh SK, de Souza D, Chu MWA, Whitlock R, Meyer SR, et al. SUPERIOR SVG: no-touch saphenous harvesting to improve patency following coronary bypass grafting (a multicentre randomized controlled trial, NCT01047449). J Cardiothorac Surg. 2019;14:85. 10.1186/s13019-019-0887-x. 31046806 PMC6498551

[8] Kachlík D, Lametschwandtner A, Rejmontová J, Stingl J, Vanek I. Vasa vasorum of the human great saphenous vein. Surg Radiol Anat. 2003;24:377–81. 12647022 10.1007/s00276-002-0067-9

[9] Taruya A, Tanaka A, Nishiguchi T, Matsuo Y, Ozaki Y, Kashiwagi M, et al. Vasa vasorum restructuring in human atherosclerotic plaque vulnerability: a clinical optical coherence tomography study. J Am Coll Cardiol. 2015;65:2469–77. 26065984 10.1016/j.jacc.2015.04.020

[10] Sugaya A, Uesugi S, Doi M, Horikoshi R, Oka N, Imada S, et al. Vasa vasorum of the no-touch saphenous vein graft observed using frequency-domain optical coherence tomography. Interdiscip Cardiovasc Thorac Surg. 2024;39:ivae167. 10.1093/icvts/ivae167. 39361309 PMC11495867

[11] Gössl M, Rosol M, Malyar NM, Fitzpatrick LA, Beighley PE, Zamir M, et al. Functional and hemodynamic characteristics of vasa vasorum in the walls of porcine coronary arteries. Anat Rec A Discov Mol Cell Evol Biol. 2003;272:526–37. 10.1002/ar.a.10060. 12740947

